# Schmallenberg virus detection in bovine semen after experimental infection of bulls

**DOI:** 10.1017/S0950268813002574

**Published:** 2013-10-09

**Authors:** W. H. M. VAN DER POEL, J. M. PARLEVLIET, E. R. A. M. VERSTRATEN, E. A. KOOI, R. HAKZE-VAN DER HONING, N. STOCKHOFE

**Affiliations:** 1 Central Veterinary Institute (CVI) of Wageningen University and Research Centre, Lelystad, The Netherlands; 2 Faculty of Veterinary Medicine, Utrecht University, The Netherlands

**Keywords:** Bovine semen, qRT–PCR, Schmallenberg virus

## Abstract

To study Schmallenberg virus (SBV) excretion in bovine semen after experimental infection, two bulls were inoculated subcutaneously with a SBV isolate (1 ml Vero cell culture 10^6^ TCID_50_). After inoculation (at day 0), semen was collected daily from both animals for 21 days and samples were tested for SBV by qRT–PCR assay. At 24 days post-inoculation both animals were subjected to necropsy and the genital organs and lymph nodes draining these organs were also tested for SBV RNA (qRT–PCR). After SBV infection both animals in the study showed viraemia (qRT–PCR) with fever and diarrhoea. SBV RNA could be detected in semen from both animals. The highest SBV RNA concentrations in semen were found in the first week (days 4–7 post-inoculation) but concentrations were relatively low (Ct values 30–39). Viable SBV was only isolated from blood samples and not from semen or genital tissues.

## INTRODUCTION

During 2011 and 2012 Europe experienced the emergence of a new arbovirus of domesticated ruminants, namely Schmallenberg virus (SBV) [1]. SBV infection causes a mild disease in adult cattle characterized by reduced milk production, pyrexia and diarrhoea. Importantly, infection of susceptible pregnant animals can be associated with abortions, and musculoskeletal and central nervous system malformations in stillborn or newborn lambs and calves. SBV was detected for the first time in November 2011 in plasma samples collected from cows displaying fever and diarrhoea near the town of Schmallenberg, Germany [2]. The first acute infections associated with SBV were reported in August 2011, while the first malformations in stillborn animals caused by this virus were detected in The Netherlands in December 2011 [3, 4]. Since 2011 SBV has spread extremely rapidly. In May 2012, eight European Union countries (Belgium, France, Germany, Italy, Luxembourg, The Netherlands, Spain, UK) had reported cases of SBV. In the summer of 2012 SBV-confirmed cases were also reported by Denmark, Finland, Poland, Sweden and Switzerland and in new areas in France, UK and Germany [5]. As of October 2012, about 6000 holdings with confirmed cases of SBV were reported by the European Food Safety Authority (EFSA) in Europe [5]. In some areas, SBV-specific antibodies have been detected in 100% of the cattle surveyed [6]. The incidence of abortions in affected farms has been variable so far. A study in 363 flocks in France showed that 15% of lambs were born dead or died within 12 h after birth or were alive after 12 h but presented deformities [7]. In ∼10% of the affected farms less than 50% of the ewes gave birth normally [7]. Thus, in some farms economic losses were substantial.

Phylogenetic analysis revealed that SBV belongs to the genus *Orthobunyavirus* within the Bunyaviridae family, a large family comprising hundreds of viruses able to infect a broad range of vertebrate and invertebrate hosts. Bunyaviruses are enveloped viruses and have a segmented single-stranded RNA genome of negative or ambisense polarity. The viral genome comprises three RNA segments referred to as small (S), medium (M) and large (L) which encode four structural proteins: the nucleocapsid protein (N); two glycoproteins (Gn and Gc); and the viral polymerase. Members of *Orthobunyavirus* encode two additional non-structural proteins, NSs and NSm. NSs is encoded by an open reading frame in the S segment overlapping the N gene while NSm is encoded by the M segment.

Bunyaviruses are significant pathogens both in humans and animals and cause a range of diseases including febrile illnesses (Oropouche virus), encephalitis (La Crosse virus) and haemorrhagic fevers (Rift Valley fever virus) [8]. SBV clusters with viruses from the Simbu serogroup, which have been associated with abortions, stillbirths and malformations (arthrogryposis–hydranencephaly syndrome) in ruminants in Asia, Africa and Oceania. Viruses from the Simbu serogroup had not been detected in vertebrates in Europe before. With the exception of hantaviruses, all bunyaviruses are transmitted by arthropod vectors.

The SBV outbreak caused major trade issues. In particular exports of live sheep and cattle and genetic products of sheep and cattle from Europe to countries outside Europe were blocked by February 2012. In December 2012 it was reported that SBV RNA was detected in bovine semen produced in European countries [9]. In an experimental infection of six calves in Germany it was proven that qRT–PCR-positive semen straw may contain infectious SBV [10]. In two of the six calves, an SBV infection could be confirmed by both real-time qRT–PCR and subsequent SBV seroconversion. In four of the six calves, neither SBV genomes nor SBV antibodies could be detected. The two semen batches which led to infection of inoculated animals had threshold cycle (Ct) values of 26·4 and 34·2, respectively. Based on those data, it was concluded that samples with a medium as well as with a low viral genome load (Ct values >30) can potentially be infectious for cattle. Currently, to declare semen free of SBV, it is advised to test semen samples for the presence of SBV RNA using an approved RNA extraction and qRT–PCR method, unless the semen was produced before June 2011 or the bull was tested SBV-specific antibody negative at least 28 days after semen production [11].

To obtain a better insight in SBV excretion in bovine semen after SBV infection, two bulls were infected experimentally and excretion of SBV in semen was studied over a period of 3 weeks. In addition at 24 days post-infection (p.i.) the bulls were necropsied and genital organs were tested for SBV.

## METHODS

### Experimental infection

The animal experiments were approved by the Ethics Committee for Animal Experiments of Utrecht University, in accordance with legislation of The Netherlands and the European Union. The two bulls (identification nos. 6361 and 6488) were 15-month-old Holstein Friesians, kindly provided by the CRV International Enterprise in the Field of Cattle Improvement. Both bulls were tested to be free of SBV-specific antibodies by virus neutralization test according to Loeffen *et al*. [12]. From the beginning of the study the two SBV-seronegative bulls were housed in a stable with a closed ventilation system, which was kept free of *Culicoides* midges by filtering the air at inlets. Both animals were inoculated subcutaneously on day 0 with Vero-cell cultured SBV which was isolated from the blood of an acutely infected cow in August 2011 in The Netherlands (1 ml supernatant, cleared by centrifugation, 500 ***g*** for 15 min, passage of two Vero cells, virus titre 10^6^ TCID_50_). This Vero cell-cultured virus was shown to be virulent by inoculation in sheep and comparison with an SBV viraemic blood inoculum. Viraemia and clinical outcomes were the same for both inocula (data not shown).

### Clinical observations and sampling

The two bulls were clinically examined daily until day 21 p.i. Every day rectal temperatures were measured and feed uptake and overt clinical signs were recorded. Blood samples were collected three times a week post-infection and semen samples were collected daily by allowing the bulls mount a dummy and using an artificial vagina. Semen samples were directly transferred to at least three different tubes: undiluted aliquots, 1:10 diluted semen in Tris (Tris-hydroxymethyl-aminomethane), 20% egg-yolk extender aliquots, and 1:10 diluted semen in fetal calf serum (FCS) aliquots. Used FCS originated from Brazil and was collected in 2010/2011 before the introduction of SBV in Europe. Blood was collected in tubes with and without EDTA to obtain whole blood as well as serum samples (900 **g**, 10 min). All samples were stored at −70° C until testing. At day 24 p.i. both bulls were necropsied and genital tissues were sampled. This included prostate glands; testicles (dorsal, ventral and central, left and right); bulbo urethralis glands; epididymis (head, body and tail, left and right) and mesenteric lymph nodes, ileocaecal lymph nodes and inguinal lymph nodes (also stored at −70° C until testing).

### Testing of samples

All samples were tested for SBV RNA by qRT–PCR as described by Bilk *et al.* [13], with minor modifications. SBV RNA was extracted from semen using a Trizol-based extraction protocol as described by Vanbinst *et al.* [14], with minor modifications. In each qRT–PCR run controls included a single SBV weak positive semen sample and multiple negative template controls. To check for inhibitory conditions, each test sample was tested with and without an internal amplification control (*β*-actin). Semen samples with extender (1:10 Tris/20% egg yolk) and without extender (undiluted aliquots) were tested in triplicate. Samples showing a Ct value of ⩽40·0 in two or three amplification runs were regarded as positive. SBV-specific antibody development was tested using the virus neutralization test as described by Loeffen *et al*. [12]. All samples showing Ct values <35 were subjected to virus isolation on Vero cell monolayers as described by Hulst *et al*. [15]. The same virus as in the inoculum (same titre) was used as positive control.

## RESULTS

**Fig. 1:**
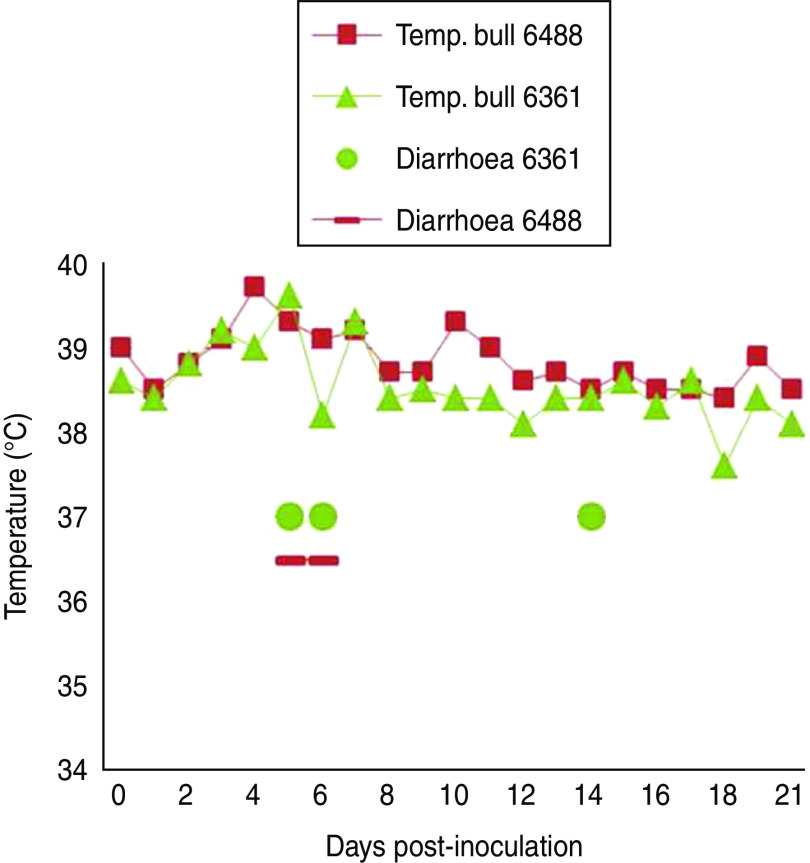
[*colour online*]. Clinical observations, body temperatures and diarrhoea, in two bulls (nos. 6361 and 6488) after inoculation with Schmallenberg virus (subcutanous injection with 1 ml SBV Vero cell culture supernatant, titre 10^6^ TCID_50_).

**Fig. 2:**
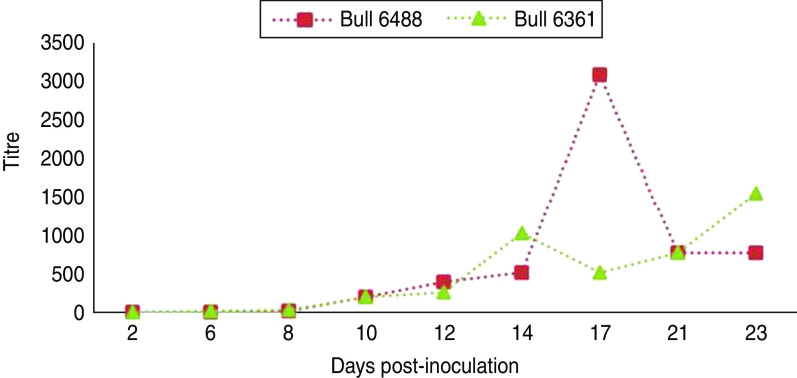
[*colour online*]. Schmallenberg virus-specific antibody development in two bulls after inoculation (day 0) with Schmallenberg virus, measured using a virus neutralization test as described by Loeffen *et al*. [12].

**Fig. 3:**
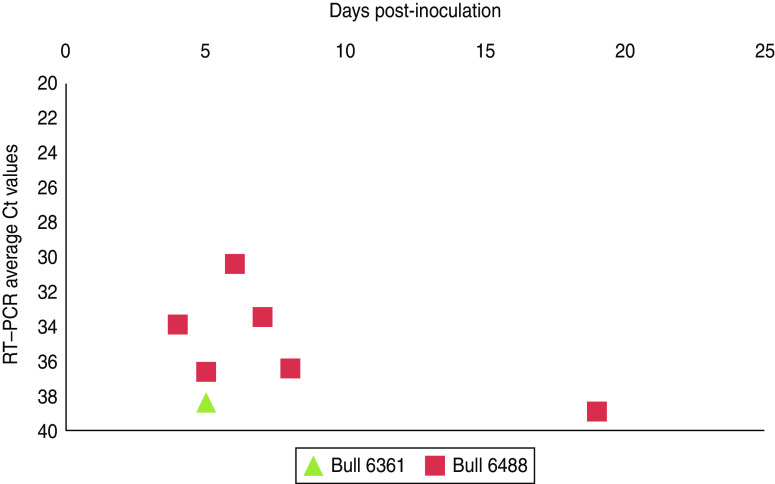
[*colour online*]. Schmallenberg virus RNA detection (RT–qPCR values) in semen samples collected from two experimentally infected bulls from day 0 until day 21 post-infection. Semen samples with extender (1:10 Tris/egg yolk 20%) (see also Table 1).

Experimental SBV infection resulted in mild clinical disease in both inoculated bulls. Both of the animals showed temperatures >39°C (days 3–7 p.i.) and some diarrhoea (days 5 and 6 p.i.) in the first week after infection (Fig. 1). SBV RNA was detected in whole blood and serum at days 2–4 p.i. After SBV infection, the development of specific antibody could be demonstrated in both animals (Fig. 2). In semen, SBV RNA was detected with the highest levels during days 4–7 p.i. (Table 1, Fig. 3), five consecutive days in one bull and at one occasion in the other. Scattered low concentration positives were found in weeks 2 and 3 after inoculation in single amplification runs (data not shown). qRT–PCR inhibitory conditions were observed in a few undiluted test samples as the internal control (*β*-actin) was not detected or showed a relatively high Ct value. After tenfold dilution and retesting, the inhibitory conditions in these samples diminished. To give an impression of the variability of the test results standard deviations are included in Table 1. SBV RNA was not detected in the genital tissues after necropsy but SBV RNA was detected in the mesenteric and inguinal lymph nodes (Table 2) that drained these tissues. SBV RNA concentrations in semen samples were relatively low (Ct values 30–39) (Table 1). SBV RNA concentrations were higher in whole blood and serum (days 2–4, Ct values 27–31) and in the lymph nodes (day 24, Ct values 29–39) (Table 2). SBV was only isolated in cell cultures from blood samples at day 3 p.i. SBV could not be isolated in Vero cells from any of the semen samples or the tissue samples, whereas positive controls were positive.

**Table 1.** Positive detections of Schmallenberg virus RNA in semen samples produced by two bulls (nos. 6361, 6488) after experimental infection

Day post-infection	Bull 6361				Bull 6488			
	Semen without extender	s.d.	Semen with extender (1:10 Tris/egg yolk 20%)	s.d.	Semen without extender	s.d.	Semen with extender (1:10 Tris/egg yolk 20%)	s.d.
0	—	—	—	—	—	—	—	—
2	—	—	—	—	—	—	—	—
3	—	—	—	—	—	—	—	—
4	—	—	—	—	37·23	(2·03)	33·87	(0·49)
5	—	—	38·37	(0·15)	n.d.	—	36·62	(1·68)
6	—	—	—	—	30·82	(0·24)	30·31	(1·54)
7	—	—	—	—	35·98	(2·07)	33·43	(1·55)
8	—	—	—	—	—	—	36·41	(1·41)
9	—	—	—	—	—	—	—	—
10	—	—	—	—	—	—	—	—
11	—	—	—	—	—	—	—	—
12	—	—	—	—	—	—	—	—
13	—	—	—	—	—	—	—	—
14	—	—	—	—	—	—	—	—
17	—	—	—	—	—	—	—	—
19	—	—	—	—	—	—	38·86	(0·33)
21	—	—	—	—	—	—	—	—

s.d., Standard deviation; n.d., not determined.

Average Ct values of three qRT–PCR amplification reactions of semen samples with and without extender (1:10 Tris/egg yolk 20%). Only samples showing two Ct values ⩽40·0 were considered positive. For further details see Materials and Methods section.

**Table 2.** Schmallenberg virus RNA detections (RT-PCR Ct values) in genital tissues and lymph node samples

Tissue	Bull 6361	Bull 6488
Mesenteric lymphnodes	38·7	30·2/29·4
Ileocaecal lymph node (right)	—	—
Ileocaecal lymph node (left)	—	—
Inguinal lymph node (right)	36·5	—
Inguinal lymph node (left)	37·9	37·0
Prostate; testicle (dorsal, ventral and central/left and right)	—	—
Bulbo urethralis (left and right)	—	—
Epididymis (head, body and tail, left and right)	—	—

## DISCUSSION

To obtain a good insight in SBV excretion in bovine semen after SBV infection, two bulls were experimentally infected, and excretion of SBV was studied over a period of 3 weeks. Only two bulls were studied but testing of produced semen resulted in SBV RNA detections in both animals with highest (semi-quantitative) levels at days 5–6 p.i. (Table 1, Fig. 3). In fact, most virus detections were made in one bull and mostly during viraemia. In the second bull there was just a single positive detection also during viraemia. SBV RNA was not detected in any of genital organs at 24 days p.i. but SBV RNA was detected in lymph nodes lying in the proximity of the genital organs.

Although semi-quantitative qRT–PCR testing showed the highest levels of SBV RNA excretion in semen in the first week after inoculation, scattered low concentration positives were found in weeks 2 and 3 after inoculation in single amplification runs. This latter observation may have been due to the fact that the concentrations of RNA in the tested semen samples were very low and close to the detection limit of the assay. Moreover, a variation in the level of virus excretions may have promoted this observation. In case such variation really occurs this might conceal intermittent excretions of low levels of RNA in semen. On the other hand this would mean that intermittent excretion cannot be excluded based on this study. In the PCR amplification reactions of a few undiluted samples, qRT–PCR inhibitory conditions were observed despite the fact that the qRT–PCR protocol was validated (using spiked semen samples) for semen with and without extender. After tenfold dilution and retesting, the inhibitory conditions in these samples diminished. By testing all semen samples with extender (1:10 Tris/egg yolk 20%) and without extender in triplicate, the variability in the test Ct values, especially around the detection limit of the assay, could be anticipated. In this way reliable outcomes could be obtained for all samplings.

Virus isolations in Vero cell cultures did not result in propagation of SBV from any of the semen samples or the tissue samples, whereas the virus was isolated from blood samples shortly after inoculation. This means that the inoculation was effective but viable SBV could not be detected in any of the semen samples or tissue samples. In a comparable study by Parsonson *et al*. [16], eight bulls were infected with Akabane virus and semen samples were tested for virus by inoculation of tissue cultures and by subcutaneous injection of susceptible cattle. In that study viable virus was also not detected in any of the semen samples. Although the Vero cell culture inoculum virus was checked for its virulence, the fact that virus was used that had been passaged in cell culture may have influenced virus excretion patterns. Cell culture adaptation may have changed the characteristics of the used SBV strain compared to ‘wild-type’ strains.

Surprisingly, SBV RNA was not detected in the genital organs at 24 days p.i., but virus RNA was detected in lymph nodes in the proximity of the genital organs. This could mean that SBV is not targeting genital tissue cells or, in the experiment, had been cleared completely from these cells at 3 weeks p.i. qRT–PCR analyses of sperm cells and seminal fluids separately showed higher levels of SBV RNA in the cell fraction (data not shown) compared to seminal fluids, but this could simply be due to attachment to cells instead of infection of these cells. Different types of cells were not tested separately in this study. Positive RNA detections in lymph nodes might represent degraded SBV cleared via the lymph system.

Both of the semen-producing bulls in the study showed a clear SBV-specific antibody development as demonstrated by virus neutralization test. In both of the animals SBV-specific antibody could be detected by virus neutralization test at 10 days p.i. One bull (6488) showed a relatively high titre at 17 days p.i.; however, this is not out of the ordinary compared to titres of other animals after infection.

The observations from this study indicate that SBV RNA can be excreted in bovine semen. This suggests that bovine semen might be contaminated with viable virus. Although viable virus was not detected in semen in this study, in an earlier study by others, infectivity of semen from SBV RNA-positive straw was reported after subcutaneous injection in calves, even in specimens with relatively low concentrations of SBV RNA [10]. In view of that it could be advised to test semen produced by SBV antibody-positive bulls by qRT–PCR [11]. However, at present it is not clear if the detected low concentrations of SBV RNA are associated with infectious virus. Moreover, even in the case of contamination of semen with infectious SBV it still needs to be elucidated if infectious virus can be transmitted to susceptible cows at service or by insemination. A study on SBV infection after insemination with qRT–PCR-positive semen straw will be needed to answer this question.
